# Resilience after the quake: life engagement and humor as pathways to trauma recovery

**DOI:** 10.3389/fpsyg.2026.1791041

**Published:** 2026-02-26

**Authors:** Şıhmehmet Yiğit, Oğulcan Usuflu, Mehmet Behzat Turan, Sevim Kır, Hakkı Ulucan, İbrahim Dalbudak, Osman Pepe, Gül Bahar Bayıroğlu

**Affiliations:** 1 Department of Sports Management, School of Physical Education and Sports, Osmaniye Korkut Ata University, Osmaniye, Türkiye; 2 Department of Physical Education and Sports, Institute of Health Sciences, Erciyes University, Kayseri, Türkiye; 3 Department of Recreation, Faculty of Sports Sciences, Erciyes University, Kayseri, Türkiye; 4 Department of Sports Management, Faculty of Sports Sciences, Erciyes University, Kayseri, Türkiye; 5 Department of Sports Management, Faculty of Sports Sciences, Uşak University, Uşak, Türkiye; 6 Department of Sports Management, Faculty of Sports Sciences, Süleyman Demirel University, Isparta, Türkiye

**Keywords:** earthquake, education, humor, life engagement, physicaleducation, psychological resilience, teacher, trauma

## Abstract

**Background:**

Earthquakes are frequently associated with elevated levels of psychological trauma, particularly among vulnerable groups. In this context, understanding the conceptual pathways and relational processes through which individuals cope becomes an important focus for research.

**Objective:**

This study investigates the serial mediation role of life engagement and coping humor in the link between psychological resilience and trauma among physical education and sport teachers affected by the earthquake, aiming to clarify how individual and psychosocial resources contribute to post-trauma adaptation.

**Methods:**

The study was conducted with 689 physical education and sport teachers affected by the earthquake. Data were collected using the Brief Resilience Scale (BRS), the Life Engagement Scale (LES), the Coping Humor Scale (CHS), and the Post Earthquake Trauma Level Determination Scale (PETLDS). Statistical analyses were performed with SPSS v22. The relationships among the variables were examined using Pearson correlation and regression analyses, and serial mediation associations were tested using the PROCESS Macro v3.5 (Model 6) with 5,000 bootstrap samples.

**Results:**

Psychological resilience was negatively associated with post-earthquake trauma in the serial mediation model (β = −0.736, *p* = 0.030). Life engagement and coping humor were associated with this relationship through statistically significant indirect pathways, as indicated by bootstrap confidence intervals that did not include zero [a1b1 = −0.110, 95% CI (−0.231, −0.018); a2b2 = −0.077, 95% CI (−0.183, −0.004); a1d1b2 = −0.033, 95% CI (−0.082, −0.003)]. These findings indicate that resilience, life engagement, and coping humor are related through theoretically consistent indirect associations within the proposed serial mediation framework.

**Conclusion:**

Psychological resilience, life engagement, and coping humor were associated with individuals’ trauma experiences following the earthquake. The findings indicate that these factors constitute relevant psychosocial resources in post-disaster contexts; however, their potential implications should be interpreted cautiously, given the study’s cross-sectional design.

## 1 Introduction

### 1.1 Earthquake

Nature has hosted some of the most devastating disasters in human history, and earthquakes rank among the most destructive natural events, caused by tectonic plate movements within the Earth’s crust (He et al., 2025; Edemen et al., 2023). Earthquakes, which occur frequently in many parts of the world, have become inevitable in certain regions. Throughout history, large-scale earthquakes have resulted in the deaths of hundreds of thousands of people, injuries to millions, and extensive destruction (Zhang et al., 2014). Thus, earthquakes are not limited to causing physical damage; they also profoundly affect social structures.

The consequences of earthquakes extend beyond their immediate impact, as they can also trigger secondary natural events. Major earthquakes can disrupt ecosystems by causing sudden landform changes, vegetation loss, and landslides (Fan et al., 2021; Huang and Li, 2014). Moreover, earthquakes occur multiple times a year and can directly or indirectly affect the lives of millions of people (Dong and Shan, 2013; Vuran et al., 2025; Pang et al., 2025). This situation, which affects all segments of society, transforms individuals’ life experiences on both physical and psychological levels (Bozkurt et al., 2023). The destruction caused by fault line movements not only harms living and non-living entities but also leads to psychological responses such as intense anxiety, fear, and Traumatic Stress Disorder (Akça Taşçı and Özsoy, 2021). In this respect, earthquakes seriously threaten mental health and social cohesion in Türkiye (Açık Yavuz et al., 2025). In this context, it is considered a global natural event threatening human and environmental security.

### 1.2 Earthquakes in Türkiye and their physical effects

Due to its location within the Mediterranean Alpine Himalayan seismic belt, Türkiye experiences approximately 20% of the world’s earthquakes and encounters at least one magnitude between 5.0 and 6.0 each year (AFAD, 2018). This geological reality leads to structural destruction and large-scale socioeconomic impacts (AFAD, 2023).

Throughout history, Türkiye has witnessed many devastating earthquakes. The 17 August 1999 Marmara Earthquake, with a magnitude of 7.4, resulted in tens of thousands of deaths, damage to hundreds of thousands of buildings, and left permanent marks on the socio-economic structure of the Marmara Region (Utkucu et al., 2011; Südaş, 2004). Subsequently, the 23 October 2011 Van Earthquake, with a magnitude of 7.2, caused severe destruction in Van and Erciş; hundreds lost their lives, and thousands of buildings became unusable (Deniz et al., 2017). More recently, the 30 October 2020 İzmir Earthquake, with a magnitude of 6.9, caused significant structural damage, particularly in the Bayraklı and Bornova districts, resulting in 117 deaths and more than 1,000 injuries (Eyler et al., 2022).

In particular, the earthquakes that occurred on February 6, 2023, were recorded as among the most devastating disasters in Türkiye’s history. Two major earthquakes, with moment magnitudes (Mw) of 7.7 and 7.6, struck 11 provinces, rupturing a fault line approximately 350 km long and affecting a total area of 110,000 km^2^ (Hussain et al., 2023; AFAD, 2023; World Health Organization [WHO], 2023). As a result of the disaster, 14 million people were directly affected; approximately 50,000 people lost their lives; 107,000 were injured; 298,000 buildings were severely damaged; and 220,000 businesses were forced to close. Additionally, 3.3 million people were displaced (International Labour Organization [ILO], 2023).

### 1.3 Psychological effects of earthquakes

Earthquakes not only lead to physical and economic losses but also lay the groundwork for long-term psychological effects and mental trauma among survivors (Zhou et al., 2019; Bulut, 2023; Karbeyaz, 2023). Post-earthquake stress reactions often include somatization, obsessive-compulsive symptoms, depression, anxiety disorders, and various other psychopathological manifestations (Adhikari Baral and K.C, 2019; Kawahara et al., 2020). Suicidal ideation resulting from traumatic experiences further highlights the severe impact of disasters on mental health.

The uncertainty and losses caused by earthquakes can impair individuals’ long-term functioning. Initial reactions such as anxiety, fear, and distractibility may evolve into persistent mental health issues over time (Öztekin and Örki, 2023; Çınaroğlu et al., 2025). A heavy trauma burden can negatively affect memory, emotional regulation, and coping mechanisms, potentially resulting in more severe psychiatric disorders (Demirchyan, 2022; Anis et al., 2023; Balbay et al., 2024; Çağış et al., 2023; Yıldırım, 2023; Aykut and Soner Aykut, 2020; Sultanoğlu et al., 2025; Bölükbaşı, 2025).

Recent studies conducted in Türkiye also support these findings. For example, it was determined that the prevalence of Post-Traumatic Stress Disorder (PTSD) among individuals affected by the 6 February 2023 earthquakes was 55.2%, with a higher risk found among women, those who lost first-degree relatives, and individuals with sleep disturbances (Aslan and Önal, 2024). Similarly, depression and anxiety levels in adults affected by the earthquake were found to be significantly associated with PTSD symptoms (Filazoğlu et al., 2025). In a study conducted with university students, a positive relationship was reported between trauma level and sleep quality, with female students being particularly negatively affected (Aylaz et al., 2025). Moreover, many studies have emphasized the protective role of psychological resilience and social support on mental health after Trauma (Kizilhan et al., 2024; Canlı and Yılmaz, 2024; Açık Yavuz et al., 2025).

Beyond these clinical and field-based findings, it is also necessary to focus on the theoretical dimension of traumatic processes. Modern psychotraumatology and the theory of dissoanalysis provide an important framework for explaining the effects of trauma at both individual and societal levels. Indeed, it has been emphasized that mass traumas such as earthquakes can particularly predispose children to dissociative disorders and PTSD, making structured mental health interventions critically important (Öztürk et al., 2023a). The study by Öztürk and Derin (2023) reveals that dissociative disorders leave traces not only at the individual but also at the societal level, showing that trauma undermines psychosocial integrity through intergenerational transmission. Similarly, Öztürk et al. (2023b) highlight that traumatic events disrupt identity, memory, and self-construction. In contrast, Derin and Öztürk (2023) indicate that societal trauma agents such as war and terrorism lead to cycles of collective trauma through mass oppression, dysfunctional parenting styles, and impaired family structures. These theoretical approaches provide a strong foundation for understanding the multilayered nature of psychosocial effects following disasters and for evaluating protective factors such as coping humor, life engagement, and psychological resilience. Individuals’ coping with such psychological impacts depends on effectively utilizing their internal resources. At this point, psychological resilience is an important protective factor in post-traumatic recovery.

### 1.4 Psychological resilience

Psychological resilience is an individual’s capacity to cope with challenges, recover from traumatic experiences, and even emerge stronger from these processes (Rutter, 2007; Marici, 2015). This concept encompasses not only overcoming adversities but also the ability to adapt to demanding experiences and demonstrate recovery.

Various researchers describe psychological resilience as an individual’s ability to recover, return to previous levels of functioning, and adapt following traumatic events (Masten and Coatsworth, 1998; Earvolino-Ramirez, 2007; Basım and Çetin, 2011). This process addresses existing adversities and contributes to maintaining healthy functioning. Indeed, psychological resilience refers to activating protective factors in the face of developmental or social risks, enabling individuals to develop positive adaptive behaviors (Winders, 2014; Güloğlu and Karaırmak, 2010).

Masten (2014) considers psychological resilience as an individual’s ability to adapt adequately to challenges throughout the developmental process. In contrast, Üzar Özçetin and Hiçdurmaz (2017) and Korkut-Owen et al. (2017) view it as the capacity to recover quickly and return to everyday life after demanding life events. Psychological resilience is not merely an immediate response but a dynamic process that continues throughout life, strengthening with new experiences (Aydoğan and Eryiğit Madzwamuse, 2020).

Similarly, Fraser et al. (1999) define psychological resilience as the ability to adapt to unexpected situations and remain positive. Earvolino-Ramirez (2007) emphasizes the individual’s capacity to recover after adversities such as illness or depressive feelings. Block and Kremen (1996) also describe psychological resilience as the adaptive and coping abilities exhibited in response to negative experiences. In this context, the emergence of psychological resilience is often triggered by traumatic experiences such as the loss of a loved one, serious illnesses, divorce, poverty, or natural disasters (Karaırmak and Siviş Çetinkaya, 2011; Güloğlu and Karaırmak, 2010).

In conclusion, psychological resilience enables individuals to activate their protective factors and adapt, recover, and continue positive development despite encountering risky and traumatic situations. This process involves overcoming challenges, reinterpreting life, and maintaining and enhancing future-oriented goals. Therefore, psychological resilience is fundamental in strengthening individuals’ connection to life and making their existence more meaningful after traumatic experiences. In this regard, it is closely associated with life engagement, which explains individuals’ sense of life satisfaction and active participation in life.

### 1.5 Life engagement

Life engagement is an individual’s ability to attribute meaning and purpose to life, which enables resilience in the face of adversity (Uğur and Akın, 2015). Maintaining a sense of existential purpose helps mitigate the psychological collapse that might otherwise result from damage and trauma (Tazegül, 2018). In the context of developmental crises, setting realistic future goals and aligning them with personal values enhances motivation and offers protection against psychological distress (McKnight and Kashdan, 2009; Eryılmaz, 2012).

Life engagement is key to emotional recovery by fostering the search for meaning following traumatic experiences (Eryılmaz, 2012). Indeed, recent studies conducted in Türkiye after earthquakes also support this process. It was found that among individuals affected by the 6 February 2023 earthquakes, as levels of meaning in life and hope increased, symptoms of Post-Traumatic Stress Disorder (PTSD) decreased, and the feeling of hope played a partial mediating role in the relationship between PTSD and meaning in life (Açık Yavuz et al., 2025). Similarly, the study by Canlı and Yılmaz (2024) revealed that the relationship between earthquake anxiety, death anxiety, and psychological resilience demonstrated the role of Life Engagement in strengthening mental health. Moreover, in the study of Karakış and Karaaziz (2024), earthquake anxiety, depression, stress, and anxiety were found to be negatively associated with psychological flexibility. It was emphasized that individuals with high psychological flexibility were less adversely affected by traumatic events and managed their mental recovery processes more effectively. Thus, incorporating life engagement into post-disaster psychosocial support programs is a crucial intervention strategy that strengthens mental health and increases resilience to future stressors. In this context, an important factor supporting life engagement is humor, which serves as a coping strategy that enhances emotional resilience.

### 1.6 Coping humor

In the face of traumatic experiences, individuals develop various defense mechanisms to maintain internal balance and strengthen their psychological resources. Among these, humor stands out as a powerful strategy that helps reframe stressful situations, reduce negative emotional burdens, and positively influence the perception of traumatic events (Öz and Hiçdurmaz, 2010; Deniz and Satıcı, 2017). Imagining humorous scenarios or creating jokes enables individuals to establish psychological distance from the distressing experience (Yerlikaya, 2009).

Humor enhances cognitive and emotional flexibility by helping individuals adopt more adaptive perspectives. Humorous elements that evoke positive emotions facilitate coping with stress and foster a positive emotional reservoir, contributing to long-term psychological resilience (Shoji et al., 2021; Kuiper and McHale, 2020). Therefore, the importance of Coping Humor increases in the context of mass traumas such as earthquakes. Although studies in this field are limited, existing findings shed light on the potential of humor to reduce post-earthquake stress, strengthen social bonds, and support recovery. Moreover, Coping Humor is an effective psychosocial strategy for alleviating trauma’s adverse effects and enhancing individuals’ resilience in the face of challenging life conditions.

This aspect of Coping Humor, which enhances individual resilience, becomes particularly significant in educational settings. In post-trauma processes, teachers are not merely transmitters of knowledge but also supportive figures contributing to students’ psychosocial recovery. Although important responsibilities fall upon all stakeholders in education (Basnet, 2020; Gezer and Şahin, 2022), physical education teachers, in particular, play a vital role in fostering both motor and emotional skills of students through the trust-based close relationships they establish in physical learning environments (Yucekaya et al., 2023). Movement-based methods reinforce the reflex to respond appropriately during sudden shaking, while group activities strengthen student solidarity and team spirit. Therefore, the expertise of physical education teachers serves as a critical bridge, not only in practical disaster drills but also in supporting students’ stress management and psychological recovery.

### 1.7 The present study

This study takes a holistic approach to the post-disaster psychological recovery processes of physical education and sport teachers. It examines the interactions among psychological resilience, life engagement, and coping humor factors, which have been largely overlooked in the literature, within the framework of a serial mediation model.

The existing literature has highlighted the decisive role of psychological resilience in individuals’ ability to cope with traumatic processes (Masten, 2014; Rutter, 2007; Üzar Özçetin and Hiçdurmaz, 2017; Yazıcı Çelebi, 2020; Vangölü and Tanhan, 2025; Helvacı Özkara and Yılmaz, 2025). Psychological resilience is defined as an individual’s capacity to adapt to challenging life experiences and emerge stronger from them, while the stress- and anxiety-reducing effects of humor have also been demonstrated in various studies (Shoji et al., 2021; Kuiper and McHale, 2020; Saul, 2022). Similarly, the psychological and physiological repercussions of traumatic experiences have been examined in detail in numerous studies (Aykut and Soner Aykut, 2020; Kino et al., 2020; Demirchyan, 2022; Tahernejad et al., 2023; Çağış et al., 2023; Anis et al., 2023; Zhou et al., 2019; Akış and Korkmaz Yaylagül, 2021; Mayer, 2009; Çifçi and Kılınç, 2024; Balbay et al., 2024). However, a notable point is that most of these studies have approached the variables independently. Existing research on teachers has generally examined psychological resilience but has predominantly used univariate, limited approaches. Therefore, a model systematically testing the sequential mediating role of life engagement and coping humor in the relationship between resilience and post-traumatic stress does not exist in the literature. This theoretical gap constitutes the most critical void the present study aims to fill.

Furthermore, examining post-trauma recovery solely at the individual level has led to the neglect of teachers’ professional resilience, their interactions with students, and the psychosocial climate within educational settings. The selection of physical education and sport teachers, particularly in the post-disaster educational context, is based on their critical role in developing students’ physical skills and supporting their psychosocial resilience (Mutch, 2015). Integrating humor into pedagogical processes and trust-based student relationships positions these teachers in a privileged role during the post-trauma recovery process.

In conclusion, this study provides original contributions to the literature at theoretical and practical levels. Theoretically, examining psychological resilience, life engagement, and coping humor within a serial mediation model offers a multidimensional and mechanistic perspective on post-trauma recovery processes. The findings are expected to inform the restructuring of in-service training programs, the development of school-based resilience strategies, and efforts to enhance the visibility of psychological preparedness within disaster management frameworks for teachers. In this regard, the present study introduces an innovative approach that holistically addresses how teachers reconstruct their psychological resilience after disasters, maintain their connection to life, and use humor as a functional coping strategy.

### 1.8 Theoretical framework and mediation pathway

The present study is grounded in resilience theory and a theoretical process perspective, which posits that psychological constructs influence outcomes not in isolation but through interrelated and sequential pathways (Fritz and MacKinnon, 2007; Hayes, 2013). Within this framework, mediation analysis provides a methodological approach to examining how the association between an independent variable and an outcome variable is transmitted through one or more intervening variables (Baron and Kenny, 1986; Hayes, 2018, 2022). While simple mediation models focus on a single intervening mechanism, serial mediation extends this approach by allowing the examination of theoretically ordered processes in which multiple mediators operate sequentially (Preacher and Hayes, 2008). This approach offers a more nuanced understanding of the pathways through which resilience-related resources are linked to post-trauma outcomes.

In the present study, psychological resilience is conceptualized as a foundational personal resource that initiates this relational sequence. Consistent with prior literature, higher levels of resilience are expected to be associated with greater life engagement, reflecting purposeful living, commitment to meaningful goals, and active involvement in life domains (Scheier et al., 2006). Individuals with higher life engagement are, in turn, more likely to employ adaptive coping strategies when confronted with adversity, particularly coping humor, which facilitates cognitive reappraisal and emotional regulation under stress (Martin, 1996; Yerlikaya, 2009). Coping humor has been shown to function as a psychological buffer, mitigating the negative psychological impact of traumatic experiences, including those arising from large-scale natural disasters such as earthquakes (Feder and Zulli, 2024).

Accordingly, the hypothesized serial mediation model proposes that psychological resilience is indirectly associated with lower levels of post-earthquake trauma through two sequential mediators: first, greater life engagement, and second, increased use of coping humor. This stepwise pathway is consistent with contemporary psychological frameworks that emphasize meaning-making processes and adaptive emotion regulation as central mechanisms underlying resilience and trauma adaptation (Kashdan and Rottenberg, 2010; Hu and Bentler, 1999). By specifying mediators in a theoretically meaningful order, the model allows for the examination of indirect associations that emerge sequentially rather than independently, thereby clarifying complex relational patterns among psychological variables (Sobel, 1982).

By testing this theoretically ordered mediation model, the present study advances beyond simple bivariate association analyses and contributes to a process-oriented understanding of how resilience facilitates psychological adjustment following disaster exposure. In line with a theoretical process perspective, the findings are intended to illuminate interrelated pathways linking personal resources, adaptive coping processes, and trauma-related outcomes rather than to suggest isolated or static protective effects (Fritz and MacKinnon, 2007; Hayes, 2013).

Based on the theoretical framework and empirical evidence, the following hypotheses were developed:

*H1*: Psychological resilience will be negatively associated with post-earthquake trauma.

*H2*: Psychological resilience will be positively associated with life engagement.

*H3*: Life engagement will be negatively associated with post-earthquake trauma.

*H4*: Psychological resilience will be positively associated with coping humor.

*H5:* Coping humor will be negatively associated with post-earthquake trauma.

*H6*: Life engagement will mediate the relationship between psychological resilience and post-earthquake trauma.

*H7*: Coping humor will mediate the relationship between psychological resilience and post-earthquake trauma.

*H8:* Life engagement and coping humor will jointly mediate the relationship between psychological resilience and post-earthquake trauma in a serial manner.

## 2 Materials and methods

### 2.1 Research model

This study employed a relational screening model to examine the relationship between variables. Relational screening aims to identify the extent to which two or more variables change in relation to each other and assess whether a significant connection exists based on this change. This non-experimental approach provides insights into the direction and strength of the relationship between variables, enabling potential predictions (Karasar, 2016; Christensen et al., 2010).

### 2.2 Determination of sample size using Monte Carlo simulation

To determine the appropriate sample size for the current study, a Monte Carlo simulation approach was employed, which is considered robust for complex models such as serial multiple mediation (Muthén and Muthén, 2002; Fritz and MacKinnon, 2007). Unlike traditional power analyses, Monte Carlo simulations allow researchers to estimate statistical power by generating data based on specified model parameters and repeatedly testing the model under these conditions. This approach is beneficial for mediation models with multiple mediators in sequence, where analytical solutions for power can be complicated or unavailable (Preacher and Kelley, 2011).

The serial mediation model in this study examined the theoretically informed indirect associations between psychological resilience (X) and post-earthquake trauma (Y) through life engagement (M1) and coping humor (M2). The analytical procedure included the following steps:

*1. Specification of model parameters:* Regression paths among variables were defined based on prior theoretical and empirical literature (Hayes, 2018; Hayes, 2022; Tugade and Fredrickson, 2004). Standardized path coefficients were specified to reflect trim-to-moderate association magnitudes commonly reported in psychological research (β = 0.20–0.30).
*2. Simulation procedure:* Monte Carlo simulations were conducted across varying sample sizes to estimate the minimum number of participants required to achieve adequate statistical power (0.80) for identifying the proposed indirect associations.
*3. Evaluation of power:* Simulation results suggested that a minimum sample size of approximately *N* = 250 would be sufficient to identify the serial indirect associations with a power level of 0.80.

**FIGURE 1:**
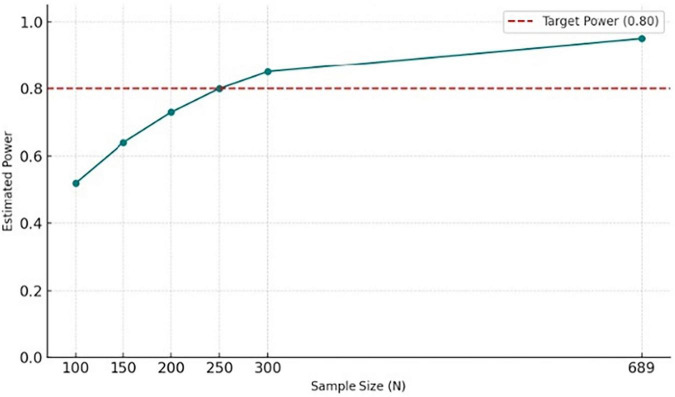
Monte Carlo simulation power analyses for the serial mediation model.

The present study included a total sample of *N* = 689, exceeding the minimum required sample size and indicating a high level of statistical sensitivity (> 0.95) for examining the proposed indirect associations. This sample size is consistent with recommendations for stable parameter estimation in mediation models and is associated with a lower risk of Type II error (Preacher and Hayes, 2008). The simulation results are presented in Figure 1.

### 2.3 Formation of the research group population

The universe refers to all individuals targeted in the study and considered in the sample selection (Şimşek, 2015). Accordingly, the universe of this study consists of physical education teachers who experienced the earthquake that occurred on February 6, 2023, and who are currently working in one of the 11 provinces affected by the disaster (Kahramanmaraş, Gaziantep, Hatay, Adana, Adıyaman, Elazığ, Diyarbakır, Kilis, Malatya, Osmaniye, and Şanlıurfa).

A convenience sampling method was preferred when selecting the sample. This non-probability sampling technique includes individuals who are easily accessible to the researcher (Altunışık et al., 2012).

The inclusion criteria for participation in the study were as follows:

1. Having resided in one of the 11 specified provinces before and after the earthquake,
2. Being physically present in these provinces at the time of the earthquake,
3. Currently working in one of these provinces.

In addition, participants outside the 11 provinces specified in the study, who completed the form incompletely or inconsistently, and who were not physical education teachers, were excluded from the sample. Participants who met these criteria and were accessible were included in the sample.

The scales prepared via Google Forms were distributed online through the Provincial Directorates of National Education in the relevant provinces during the data collection. Participation in the study was entirely voluntary. As a result, 689 physical education and sports teachers participated in the study, exceeding the targeted sample size.

The reason for choosing physical education teachers as the sample is that, after disasters, they help develop physical skills and play an important role in supporting students’ psychosocial resilience (Mutch, 2015). Their trust-based relationships with students and ability to integrate humor into pedagogical approaches place physical education teachers in a critical position within post-trauma recovery processes. Therefore, in the present study, physical education teachers were considered an appropriate sample for evaluating the serial mediating role of life engagement and coping humor in the relationship between resilience and trauma.

### 2.4 Study model

This study aims to examine the association between psychological resilience and post-earthquake trauma among physical education and sport teachers, and to explore the theoretically informed serial mediation role of life engagement and coping humor in this association.

Serial multiple mediation models are analytical frameworks used to examine indirect associations among variables through multiple mediators arranged in a theoretically specified sequence. Rather than implying causality, such models allow researchers to assess whether the associations between a predictor and an outcome may be conceptually sequentially linked through intermediary variables (Hayes, 2018; Hayes, 2022).

**FIGURE 2:**
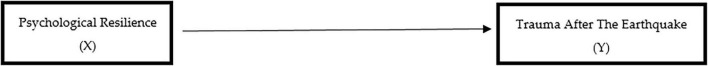
Direct association between psychological resilience and post-earthquake trauma.

**FIGURE 3:**
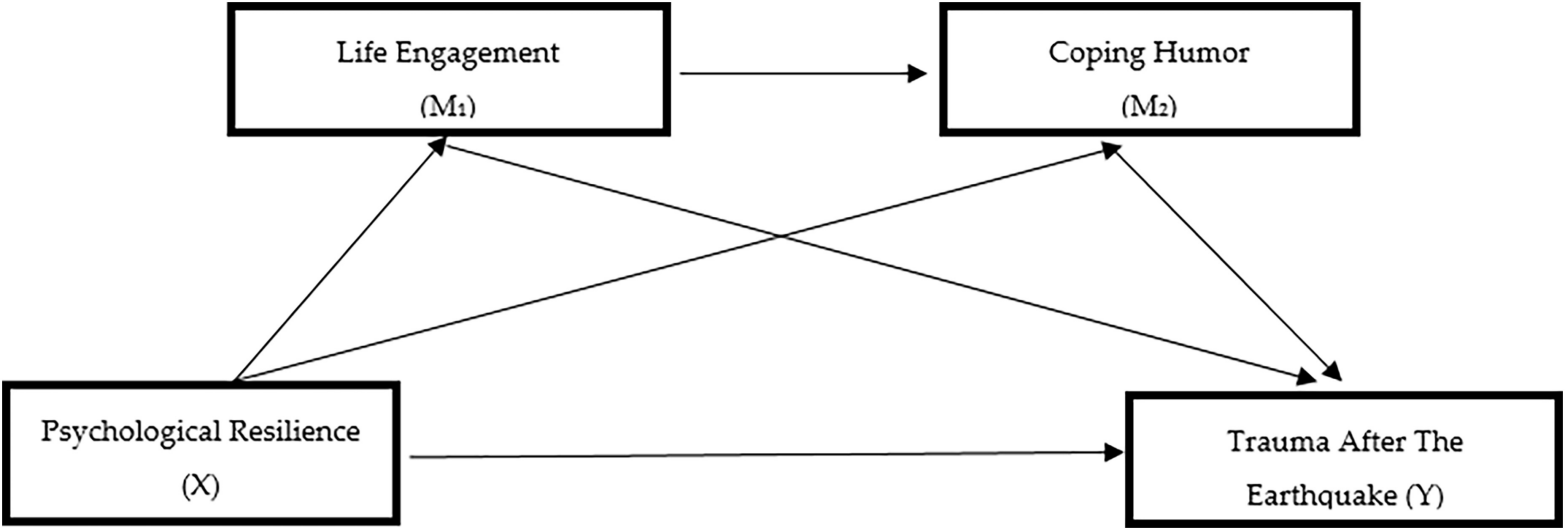
Serial multiple mediation model examining the indirect effects of psychological resilience on post-earthquake trauma through life engagement and coping humor.

Accordingly, the serial multiple mediation model examined the indirect associations of psychological resilience (X) with post-earthquake trauma (Y) through life engagement (M1) and coping humor (M2). Figure 2 presents the association between psychological resilience and post-earthquake trauma, while Figure 3 illustrates a theoretically informed serial mediation model that includes life engagement and coping humor.

### 2.5 Data collection form

#### 2.5.1 Personal information form

A personal information form comprising eight variables was prepared to collect demographic and professional data from participants. These variables include gender, age, marital status, level of education, years of professional experience, type of institution, current place of duty, and whether the participant holds an administrative position.

Table 1 shows that 305 participants (44.3%) are female and 384 participants (55.7%) are male. Regarding age groups, 131 participants (19.0%) are between 27 and 33 years old, 153 (22.2%) are between 34 and 40 years old, 199 (28.9%) are between 41 and 47 years old, and 206 (29.9%) are between 48 and 54 years old. Regarding marital status, 527 participants (76.5%) are married, while 162 (12.5%) are single. Regarding educational level, 580 participants (84.2%) hold a bachelor’s degree, while 109 participants (15.8%) hold a postgraduate degree. For professional experience, 122 participants (17.7%) have 1–10 years, 183 participants (26.6%) have 11–20 years, and 384 participants (55.7%) have 21–30 years. Regarding the place of duty, 172 participants (25.0%) work in districts, while 517 (75.0%) work in city centers. Lastly, concerning administrative duty, 299 participants (43.4%) hold administrative roles, whereas 334 participants (56.6%) do not.

**TABLE 1:** Descriptive statistics: frequency and percentage values.

Variable	Group	*N*	*f*	%
Gender	Female	689	305	44.3
	Male		384	55.7
Age	27–33	689	131	19.0
	34–40		153	22.2
	41–47		199	28.9
	48–54		206	29.9
Marital status	Married	689	527	76.5
	Single		162	12.5
Educational level	Undergraduate	689	580	84.2
	Postgraduate		109	15.8
Years of service	1–10	689	122	17.7
	11–20		183	26.6
	21–30		384	55.7
Place of duty	District	689	172	25.0
	City center		517	75.0
Administrative duty	Yes	689	299	43.4
	No		390	56.6

#### 2.5.2 The Brief Resilience Scale

The Brie Resilience Scale (BRS) was developed to measure individual resilience. The scale was developed by Smith et al. (2008) and adapted into Turkish by Doğan (2015). The scale adapted to Turkish culture consists of 6 items (e.g., “After difficult times, I can quickly recover”).

It uses a 5-point Likert-type rating system ranging from “1-Not suitable at all” to “5- Completely suitable.” Items 2, 4, and 6 are reverse-scored items on the reverse-coded scale; high scores indicate high psychological resilience. The Cronbach’s alpha internal consistency reliability coefficient was reported as 0.83.

#### 2.5.3 The Life Engagement Scale

The Life Engagement Scale was developed by Scheier et al. (2006) to assess individuals’ commitment to their life goals and was subsequently adapted into Turkish by Uğur and Akın (2015). The scale comprises six items (e.g., “I do not have many goals in my life.”) and is evaluated under a single dimension (commitment to life). It uses a 5-point Likert-type rating system ranging from “1- Strongly Disagree” to “5-Strongly Agree.” Items 1, 3, and 5 are reverse-coded. Higher scores indicate greater commitment to life goals. The construct validity of the original form of the scale was tested in different study groups. A unidimensional structure was obtained that explained between 43 and 62% of the total variance, with factor loadings ranging from 0.57 to 0.86. The internal consistency coefficient of the scale ranged from 0.72 to 0.87. In contrast, the test-retest reliability coefficients ranged from 0.61 to 0.76 (Scheier et al., 2006). In the Turkish adaptation, Cronbach’s alpha was 0.74 (Uğur and Akın, 2015).

#### 2.5.4 The Coping Humor Scale

The Coping Humor Scale, developed by Martin (1996), assesses humor as a coping strategy for stressful life events. The Turkish adaptation was carried out by Yerlikaya (2009). The scale consists of seven items evaluated on a single dimension, using a 4-point Likert-type rating system ranging from “1-Strongly Disagree” to “4-Strongly Agree.” An example item is: “When I have problems, I often lose my sense of humor.” Total scores range from 7 to 28, with higher scores indicating a greater use of humor in stress management. Internal consistency coefficients of the original scale ranged from 0.60 to 0.70, and test–retest reliability (after 2 weeks) was reported as 0.80 (Martin, 1996). The Turkish version demonstrated a Cronbach’s alpha of 0.67 (Yerlikaya, 2009).

#### 2.5.5 The Post-Earthquake Trauma Level Determination Scale

Tanhan and Kayri (2013) developed the Post Earthquake Trauma Level Determination Scale to assess individuals’ reactions to trauma following a natural disaster. The scale comprises 20 negatively worded items across five subdimensions: Behavioral Problems, Emotional Limitation, Affective, Cognitive Structuring, and Sleep Problems. It uses a 5-point Likert scale ranging from “1-Disagree” to “5-Agree.” An example item is: “I sleep less.” Total scores range from 20 to 100, with higher scores indicating greater trauma impact from the earthquake. Exploratory and confirmatory factor analyses were conducted for construct validity. The Cronbach’s alpha coefficient for the original scale was reported as 0.74 (Tanhan and Kayri, 2013).

The Cronbach alpha values obtained from participants’ responses are presented in Table 2.

**TABLE 2:** Descriptive values of the subdimensions of the scales.

Scales	Number of ıtems	Cronbach’s alpha
The brief resilience	6	0.860
The life engagement	6	0.774
The coping humor	7	0.844
The post-earthquake trauma level determination	20	0.937

When Table 2 is examined, Cronbach’s Alpha values indicate that the internal consistency coefficient for the brief resilience scale is 0.860, for the life engagement scale is 0.774, for the coping humor scale is 0.844, and for the post-earthquake trauma level determination scale is 0.937.

Cronbach’s Alpha is a reliability coefficient used to assess the internal consistency of multi-item scales. In other words, it is used to determine the extent to which the items in a scale are related to each other, that is, whether they measure the same concept. The value range is 0–1; a value of 0.70 or higher is generally considered an acceptable level of internal consistency (Nunnally and Bernstein, 1994). These values demonstrate that the participants’ data on these scales exhibit an acceptable level of internal consistency.

The fit indices from the Confirmatory Factor Analysis (CFA) were used to assess the model’s fit to the data. Considering the limits accepted in the literature, all indices were acceptable or indicated a good fit. As shown in Table 3, all scales exhibit acceptable model fit.

**TABLE 3:** Fit indices of scales.

The scales	χ ^2^/df	CFI	TLI	RMSEA	SRMR
Brief resilience	2.21	0.99	0.97	0.042	0.021
Life engagement	2.56	0.99	0.99	0.048	0.013
Coping humor	1.51	0.99	0.99	0.027	0.027
Post earthquake trauma level determination	2.90	0.99	0.98	0.058	0.057

When examining the results in Table 3, the model presents excellent fit indices for all scales, with χ^2^/df values ranging from 1.51 to 2.90, indicating reasonable to good model fit. CFI and TLI values consistently exceed 0.99, demonstrating a strong fit across the models. RMSEA and SRMR values are all within acceptable limits, further supporting the models’ adequacy. These results suggest that the Brief Resilience, Life Engagement, Coping Humor, and Post-Earthquake Trauma Level Determination scales are well-defined and have strong psychometric properties. Exploratory Factor Analysis results are provided in Supplementary Appendix A.

### 2.6 Data analysis

Data was analyzed using SPSS v22. The Kolmogorov-Smirnov test, one of the tests used to assess the normality of data distributions (Berger and Zhou, 2014), was employed. The normality results of the scores obtained in this study are presented in Table 4.

**TABLE 4:** Skewness, Kurtosis, and Kolmogorov-Smirnov Test significance level results of the participants’ scale scores.

The scales	Skewness	Kurtosis	*P*
Brief resilience	0.224	1.438	0.000
Life engagement	−0.005	−0.148	0.011
Coping humor	−0.511	0.283	0.000
Post earthquake trauma level determination	0.218	−0.195	0.000

The VIF values of the independent variables ranged from 1.024 to 1.119, and no multicollinearity was observed. The distribution of standardized residuals was normal and homogeneous. Cook’s Distance values ranged from 0.000 to 0.038, indicating that outliers did not significantly affect the regression results.

Regression Model Summary and ANOVA were conducted to examine the regression model’s explanatory power. The associations between the independent variables and the dependent variable were examined using regression coefficient tables.

When Table 4 is examined, the skewness and kurtosis values of the data fall within ± 1.5. Values within ± 2 (George and Mallery, 2010) indicate the absence of excessive deviations from normality. Consequently, the data were normally distributed and suitable for parametric tests. Pearson correlation analysis was used to examine the relationships among the study variables, and the Fisher Z transformation test was applied to compare these relationships. Regression analyses were conducted to examine the associations among psychological resilience, life engagement, coping humor, and post-earthquake trauma. To examine the serial mediation associations among psychological resilience, life engagement, and coping humor, regression analyses based on the indirect-effect approach using the bootstrap method were conducted with the PROCESS v3.5 macro. The PROCESS Macro Model 6, developed by Hayes (2013), was used to test the theoretically specified serial mediation model, with 5,000 bootstrap resamples applied. The statistical significance of the indirect associations was evaluated using 95% confidence intervals from the bootstrap analysis; intervals that did not include zero indicated statistically significant indirect associations (Gürbüz, 2019; Hayes, 2013).

## 3 Results

When Table 5 was examined, it was determined that the level of brief resilience of the physical education teachers participating in the study was 19.943 ± 2.043, life engagement was 26.781 ± 3.670, coping humor was 19.591 ± 5.219, and post-earthquake trauma was 67.790 ± 18.273.

**TABLE 5:** Descriptive statistics of the scales.

The scales	Min	Max	M ± SD
1. Brief resilience	10.00	26.00	19.943 ± 2.043
2. Life engagement	15.00	30.00	26.781 ± 3.670
3. Coping humor	7.00	28.00	19.591 ± 5.219
4. Post-earthquake trauma	21.00	96.00	67.790 ± 18.273

When Table 6 is examined, Fisher’s Z transformations for the correlations between the various scales were calculated as follows: the correlation between participants’ brief resilience and their life engagement (*r* = 0.123, *p* < 0.001) resulted in a Z score of 0.121, their coping humor (*r* = 0.125, *p* < 0.001) resulted in a Z score of 0.131, and post-earthquake trauma (*r* = −0.107, *p* < 0.001) resulted in a Z score of −0.110. The correlation between participants’ life engagement and their coping humor (*r* = 0.315, *p* < 0.001) yielded a Z score of 0.332; the correlation between participants’ post-earthquake trauma and their coping humor (*r* = −0.141, *p* < 0.001) yielded a Z score of −0.141. The correlation between participants’ coping humor and their post-earthquake trauma (*r* = −0.141, *p* < 0.001) yielded a Z score of −0.141.

**TABLE 6:** Pearson correlation coefficients for the correlations between the variables.

The scales	1	2	3	4
1. Brief resilience	1	0.123**	0.125**	−0.107**
2. Life engagement	0.123**	1	0.315**	−0.141**
3. Coping humor	0.125**	0.315**	1	−0.141**
4. Post-earthquake trauma	−0.107**	−0.141**	−0.141**	1

**p < 0.001, *n* = 474, 1- Brief Resilience, 2- Life Engagement, 3- Coping Humor, 4- Post-Earthquake Trauma.

**FIGURE 4:**
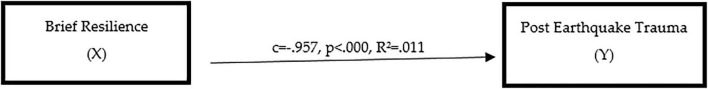
Direct@@@ association between brief resilience and post-earthquake trauma.

**FIGURE 5:**
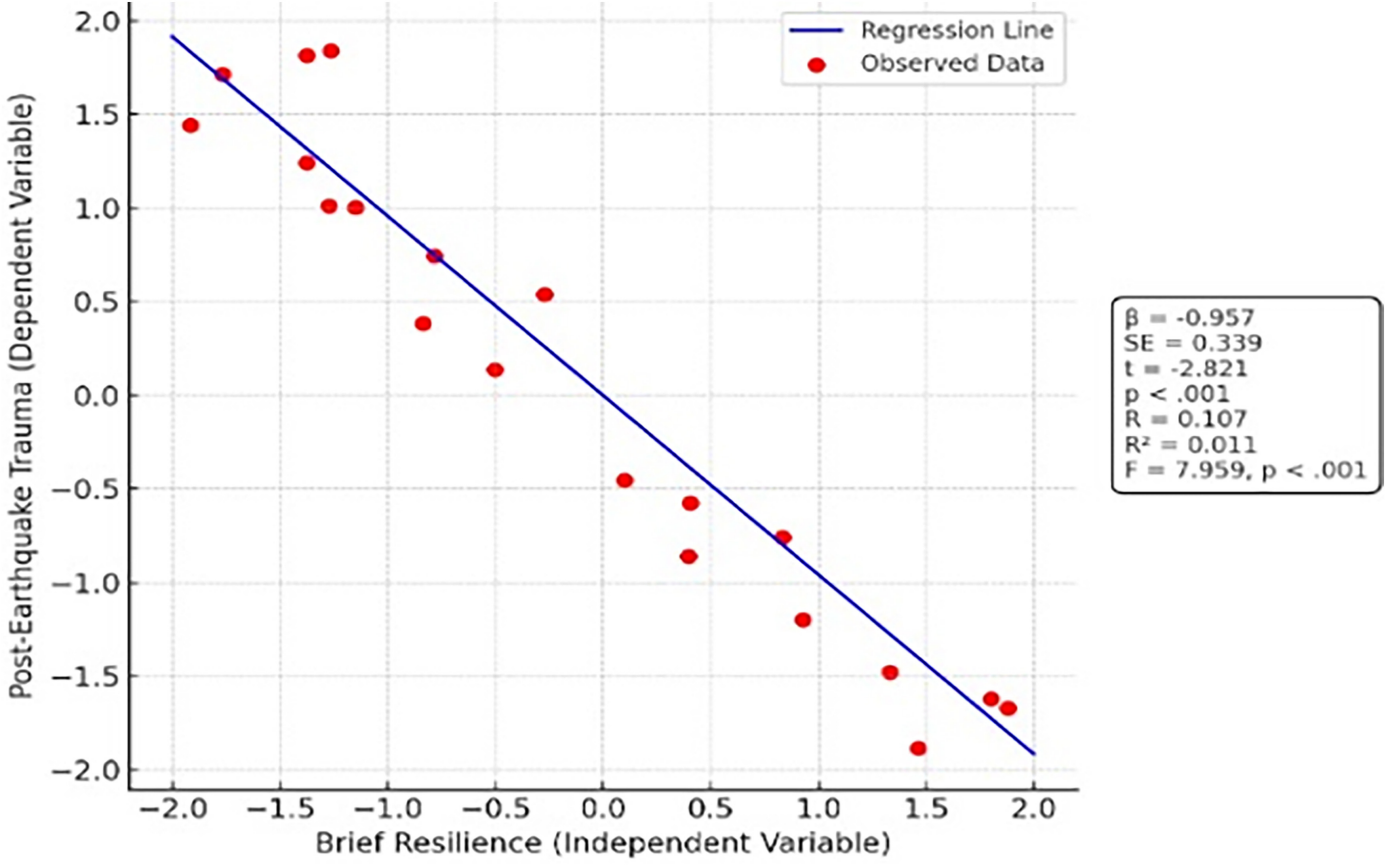
Regression model summary for the association between brief resilience and post-earthquake trauma.

When Table 7 is examined, the regression analysis indicates a statistically significant association between psychological resilience and post-earthquake trauma among physical education teachers [*F*(1, 687) = 7.959, *p* < 0.001]. As shown in Figure 4, there is a direct association between brief resilience and post-earthquake trauma. The regression model summary examining the association between brief resilience and post-earthquake trauma is presented in Figure 5. The regression coefficient was negative and statistically significant (β = −0.957, *t* = −2.821, *p* < 0.001). The model accounted for approximately 1.1% of the variance in post-earthquake trauma (*R*^2^ = 0.011).

**TABLE 7:** The association between brief resilience and post-earthquake trauma.

Variables									
Independent	Depend	β	*SE*	*t*	*p*	*R*	*R* ^2^	*F*	*p*
Brief resilience	Post-earthquake trauma	−0.957	0.339	−2.821	0.000	0.107	0.011	7.959	0.000

When Table 8 is examined, Tolerance values (> 0.89) and VIF values (< 1.12) indicate that multicollinearity is not a concern in this model. This means the predictors are sufficiently independent of one another and each provides unique explanatory power.

**TABLE 8:** Collinearity statistics of predictor variables.

Predictor	Tolerance	VIF
Coping humor	0.894	1.119
Life engagement	0.894	1.119
Brief resilience	0.977	1.024

When Table 9 is examined, Cook’s D values range from 0.000 to 0.038, with an average of 0.002. Since all values are well below the threshold of 1, no single case has an undue influence on the regression results. This indicates that the findings are robust and not driven by outliers.

**TABLE 9:** Cook’s distance statistics.

Statistic	Min	Max	Mean	SD
Cook’s distance	0.000	0.038	0.002	0.003

When Table 10 is examined, the regression model accounts for 3.7% of the variance in post-earthquake trauma (*R*^2^ = 0.037). Although the proportion of explained variance is relatively small, the model is statistically significant (*p* < 0.001). This finding indicates that the predictors included in the model are statistically associated with post-earthquake trauma levels, suggesting a theoretically meaningful relationship, even though the magnitude of the association is modest.

**TABLE 10:** Regression model summary.

Model	*R*	R square	Adjusted R square	Std. error	Sig.
1	0.192	0.037	0.033	17.973	0.000**

**p < 0.01.

When Table 11 is examined, the ANOVA results indicate that the regression model is significant overall [*F*(3, 685) = 8.737, *p* < 0.001]. This suggests that the set of predictors (Coping Humor, Life Engagement, and Brief Resilience) jointly explains a significant proportion of the variation in post-earthquake trauma, beyond what would be expected by chance.

**TABLE 11:** Analysis of variance (ANOVA) for the regression model.

Model	Sum of squares	df	Mean square	*F*	Sig.
Regression	8466.571	3	2822.190	8.737	0.000**
Residual	221267.914	685	323.019		
Total	229734.485	685			

**p < 0.01.

When Table 12 is examined, all three predictors are negative and statistically significant:

**TABLE 12:** Regression coefficients for predictors of post-earthquake trauma.

Predictor	*B*	Std. error	Beta	*T*	Sig.
(Constant)	102.527	7.939	–	12.914	0.000**
Coping humor	−0.348	0.139	−0.099	−2.507	0.012*
Life engagement	−0.494	0.197	−0.099	−2.508	0.012*
Brief resilience	−0.736	0.339	−0.082	−2.170	0.030*

*p < 0.05,

**p < 0.01.

Coping Humor (β = –0.099, *p* = 0.012): Higher coping humor is associated with lower levels of trauma.
Life Engagement (β = –0.099, *p* = 0.012): Greater life engagement predicts lower trauma levels.
Brief Resilience (β = –0.082, *p* = 0.030): Higher resilience contributes to a reduction in trauma.

These findings indicate that psychological resources such as humor, engagement in life, and resilience act as protective factors against post-earthquake trauma.

When Table 13 was examined, brief resilience was found to be positively and statistically significantly associated with life engagement (path a1) (a1 = 0.222, *t* = 8.898, *p* = 0.001). Life engagement was negatively and significantly associated with post-earthquake trauma (path b1) (b1 = −0.494, *t* = −2.508, *p* = 0.001). In addition, brief resilience was positively and statistically significantly associated with coping humor (path a2) (a2 = 0.223, *t* = 1.569, *p* = 0.001). Coping humor, in turn, was negatively and significantly associated with post-earthquake trauma (path b2) (b2 = −0.348, *t* = −2.507, *p* = 0.001). Furthermore, life engagement was found to be positively and statistically significantly associated with coping humor (path d1) (d1 = 0.431, *t* = 8.356, *p* = 0.001). When the direct association between brief resilience and post-earthquake trauma (path c’) was examined, this relationship was also found to be statistically significant (c’ = −0.736, *t* = −2.170, *p* = 0.001).

**TABLE 13:** The serial mediation role of life engagement and coping humor between brief resilience and post-earthquake trauma (*N* = 689).

	Life engagement (M_1_)				Coping humor (M_2_)				Post-earthquake trauma (Y)			
		*b*	SE	*t*		*b*	SE	*t*		*b*	SE	*t*
Outcomes												
Brief resilience (X)	a1	0.222	0.068	16.351	a2	0.223	0.093	1.659	c’	−0.736	0.339	−2.170
Life engagement (M_1_)	–	–	–	–	d1	0.431	0.052	8.356	b1	−0.494	0.197	−2.508
Coping humor (M_2_)	–	–	–	–	–	–	–	–	b2	–0.348	0.139	–2.507
Constant		22.349	1.367	16.351		3.613	2.178	1.659		102.527	7.939	12.914
		*R*^2^ = 0.015				*R*^2^ = 0.107				*R*^2^ = 0.037		
		*F*(1,687) = 10.626				*F*(1,687) = 430.870				*F*(1,687) = 8.737		
		*P* = 0.001				*P* = 0.000				*P* = 0.000		

**FIGURE 6:**
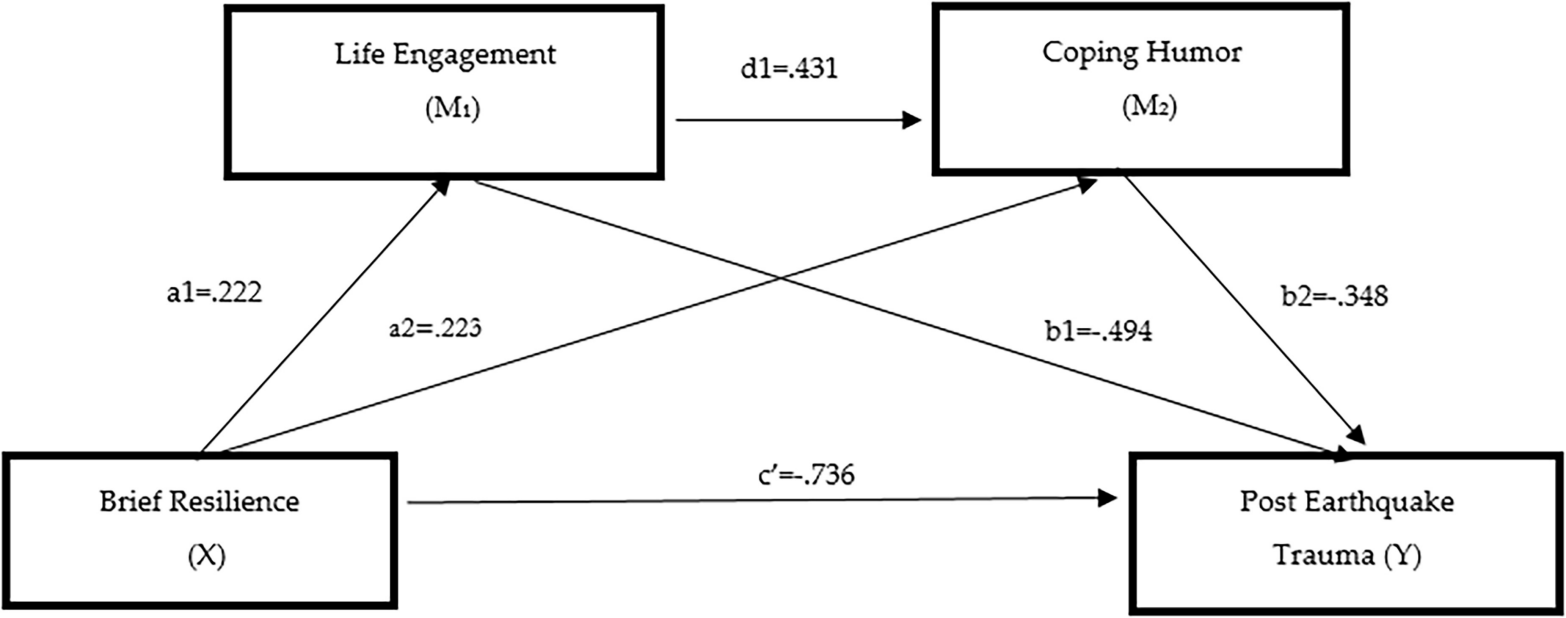
Serial mediation model illustrating the sequential associations of life engagement and coping humor in the relationship between psychological resilience and post-earthquake trauma.

As illustrated in Figure 6, a serial multiple mediation Model 6 was tested. The model includes two mediating variables, three indirect associations, and one direct association. These associations are defined as follows: the indirect association between brief resilience and post-earthquake trauma via life engagement (a1b1); the indirect association between brief resilience and post-earthquake trauma via coping humor (a2b2); and the serial indirect association between brief resilience and post-earthquake trauma via life engagement and coping humor (a1d1b2). The sum of these three indirect associations represents the total indirect association between brief resilience and post-earthquake trauma (a1b1 + a2b2 + a1d1b2).

As shown in Table 14, brief resilience was negatively and statistically significantly associated with post-earthquake trauma at the direct level [*B* = −0.221, SE = 0.087, CI (−0.411, −0.071)]. The first indirect association reflects the relationship between brief resilience and post-earthquake trauma through life engagement (brief resilience → life engagement → post-earthquake trauma). This indirect association was statistically significant (*B* = −0.110, SE = 0.054, CI [−0.231, −0.018]). The second indirect association represents the relationship between brief resilience and post-earthquake trauma through coping humor (brief resilience → coping humor → post-earthquake trauma). This indirect association was also statistically significant [*B* = −0.077, SE = 0.047, CI (−0.183, −0.004)]. The third indirect association reflects the serial relationship between brief resilience and post-earthquake trauma through life engagement and coping humor (brief resilience → life engagement → coping humor → post-earthquake trauma). This serial indirect association was statistically significant [*B* = −0.033, SE = 0.021, CI (−0.082, −0.003)].

**TABLE 14:** The indirect associations between brief resilience and post-earthquake trauma.

Indirect effects	*b*	SE	LLCI	ULCI
Total effect	−0.221	0.087	−0.411	−0.071
Ind 1	−0.110	0.054	−0.231	−0.018
Ind 2	−0.077	0.047	−0.183	−0.004
Ind 3	−0.033	0.021	−0.082	−0.003

## 4 Discussion

This study examined the associations among psychological resilience and post-earthquake trauma among physical education and sport teachers, with particular attention to the serial mediation and moderating roles of life engagement and coping humor. The study addressed the relationships between participants’ levels of psychological resilience, life engagement, coping humor, and their trauma after the earthquake. The findings revealed that these three psychosocial resources reduce trauma both directly and indirectly.

Regression analyses indicated that psychological resilience, life engagement, and coping humor significantly and negatively predicted trauma; in other words, higher levels of these resources contributed to lower trauma symptoms. Although the model explained relatively slight variance (*R*^2^ = 0.037), the findings reinforce the literature supporting the protective role of psychosocial factors in post-trauma processes (Bonanno, 2004; Masten, 2014; Tugade and Fredrickson, 2004). The findings suggest that psychological resilience, life engagement, and coping humor are psychosocial resources that are theoretically associated with individuals’ capacity to cope with traumatic experiences following a disaster.

From a theoretical perspective, psychological resilience has been conceptualized as a factor associated with more adaptive emotional and cognitive responses to traumatic experiences. Resilient individuals tend to draw on positive emotions in stressful contexts and to employ flexible cognitive appraisals, which are linked to a broader range of coping strategies (Tugade and Fredrickson, 2004; Bonanno, 2004). When considered alongside life engagement, this process may be conceptualized as individuals’ orientation toward meaningful goals and sustained involvement in valued life domains, which have been associated with more adaptive adjustment under adversity (Scheier et al., 2006; Park, 2010). Similarly, coping humor may be theoretically related to this association by supporting emotion regulation in stressful situations and facilitating social connectedness, both of which are commonly discussed in the literature as resources in trauma-related contexts (Martin, 2007; Kuiper, 2012).

The findings indicate that psychological resilience is directly and indirectly associated with trauma through its relationships with life engagement and coping humor, suggesting that these three factors function as complementary psychosocial resources within a shared conceptual pathway. Life engagement shapes the impact of traumatic experiences by strengthening individuals’ connection to life in accordance with meaning and purpose (Scheier et al., 2006; Park, 2010). However, the findings also suggest that life engagement is not always a protective factor; in some cases, it may increase individuals’ vulnerability to trauma. That is, individuals with high life engagement may be more sensitive to environmental threats and traumatic events, experiencing trauma more intensely (Şar et al., 2019; Arguvanlı Çoban and Gün Koşar, 2024).

The findings also indicate that coping humor is associated with lower levels of trauma.

Humor is conceptually linked to threat perception reframing, is associated with lower levels of stress, and is related to interpersonal support within relational pathways (Martin, 2007). Research suggests that humor is associated with lower levels of negative emotional intensity, greater use of positive reappraisal, and higher levels of perceived social cohesion in post-disaster contexts (Samson and Gross, 2012; Kuiper, 2012). The present study demonstrates that humor partially explains the relationship between life engagement and trauma, indicating that it plays a regulatory and indirect role in coping processes (Saylam and Sapancı, 2025; Kim et al., 2022; Xie et al., 2017).

Mediation analyses showed that coping humor had a statistically significant indirect association in the relationship between life engagement and trauma [indirect association.061, 95% CI (1.176, 1.742)]. This suggests that individuals with higher levels of life engagement tend to report greater use of humor when dealing with traumatic experiences, and that humor is conceptually related to trauma outcomes within this relational framework. From a theoretical standpoint, coping humor may be understood as a psychosocial resource that helps individuals navigate and regulate stressful experiences, rather than as a direct explanatory construct. In this context, humor can be viewed as a conceptual link that organizes the associations among psychosocial resources, consistent with prior research emphasizing its role in emotional regulation and interpersonal processes in adversity-related contexts (Boerner et al., 2017; Schneider et al., 2018).

Life engagement also significantly predicted trauma, indicating that psychological processes related to individuals’ life goals and search for meaning play a determining role in post-trauma responses (β = 0.401, *p* < 0.001; *R*^2^ = 0.161). The literature supports that such intrinsic values can function both protectively and, under certain conditions, as sensitivity-enhancing factors (Gökçay et al., 2024; Durmuş and Durar, 2021; Arguvanlı Çoban and Gün Koşar, 2024).

The findings indicate that psychological resilience, life engagement, and coping humor are interrelated psychosocial resources that are associated with lower levels of trauma following the earthquake. Within the proposed model, life engagement and coping humor appear to be theoretically linked to the association between psychological resilience and trauma, suggesting that these resources may operate together in shaping individuals’ post-disaster psychological experiences. Overall, the results highlight the importance of considering psychosocial resources in combination rather than in isolation when examining trauma-related outcomes.

From an intervention-oriented and contextual perspective, the present findings can be situated within dynamic, process-based therapeutic frameworks that conceptualize psychological adjustment as a flexible, meaning-oriented system rather than a static set of protective traits (Kaya, 2022). Contemporary integrative models, including process-based and contextual approaches within cognitive-behavioral traditions, highlight cognitive flexibility, meaning-making, and adaptive self-regulation as key conceptual pathways that shape how individuals navigate heightened stress and uncertainty (Hofmann and Hayes, 2019; Bonanno et al., 2015; Southwick et al., 2014). These principles conceptually align with the roles of psychological resilience, life engagement, and coping humor identified in the current model, suggesting that these resources may be understood as interrelated processes embedded within broader systems of adaptation rather than as isolated predictors of trauma-related outcomes.

Furthermore, evidence from large-scale crisis contexts, including mixed-method and population-based research on the mental health impacts of the COVID-19 pandemic, highlights how psychosocial resources operate within social, demographic, and cultural contexts, shaping differential patterns of vulnerability and coping across populations (Loades et al., 2020; Pierce et al., 2020; Hobfoll et al., 2007; Kaya and McCabe, 2022). Situating the present findings within this broader disaster and crisis mental health literature underscores the value of examining resilience-related processes beyond symptom-focused outcomes and reinforces the relevance of examining resilience beyond symptom-focused outcomes.

From a practical perspective, post-disaster psychosocial interventions may benefit from approaches that simultaneously support and cultivate these resources, rather than targeting a single factor (Southwick and Charney, 2012; Li et al., 2020; Yıldırım, 2023).

## 5 Conclusion

This study examined psychosocial resources associated with post-earthquake trauma levels specifically among physical education and sport teachers, a professional group characterized by high interpersonal demands and sustained engagement in physically and emotionally intensive educational settings. The findings indicate that psychological resilience, life engagement, and coping humor are statistically related to trauma outcomes and tend to co-occur in patterns that are theoretically consistent with adaptive coping processes. Importantly, these associations should not be interpreted as reflecting theoretical determinism or fixed protective effects; rather, they underscore how multiple psychosocial resources operate jointly in shaping individuals’ trauma-related experiences in post-disaster contexts.

At the level of physical education and sport teachers, the results suggest that coping humor may function as a regulatory and linking process within broader coping dynamics, indirectly connecting life engagement to trauma-related outcomes. Life engagement, in this context, reflects an orientation toward meaning, purpose, and goal-directed living, which may support adaptive responses to trauma while also heightening emotional sensitivity under certain conditions. These findings highlight the relevance of profession-specific intervention strategies that acknowledge both the emotional demands and the meaning-oriented nature of physical education teaching, particularly in the aftermath of large-scale disasters.

From an applied perspective, the findings point to the potential value of post-disaster interventions designed specifically for physical education and sport teachers, emphasizing the strengthening of psychological resilience, the cultivation of life meaning and engagement, and the development of adaptive coping humor skills. Such interventions may enhance educators’ capacity to manage trauma-related stress while supporting psychological wellbeing and professional functioning in school environments affected by disaster-related disruptions.

At the same time, the implications of the present findings for broader educational or occupational groups should be interpreted with caution. While the identified psychosocial processes may be relevant to other professions characterized by high interpersonal interaction and emotional labor, the current results are empirically grounded in a specific professional and cultural context. Accordingly, generalization beyond physical education and sport teachers should be considered tentative, and future research is needed to examine whether similar process-based relationships emerge across different occupational groups and disaster contexts.

Finally, although the observed effects were statistically significant, the relatively low explained variance (R^2^ values) indicates that the examined variables account for a modest proportion of post-earthquake trauma outcomes. This suggests that additional psychological, contextual, and environmental factors such as organizational support, community resources, and prior trauma exposure are likely to play an important role. Taken together, the findings support a multidimensional and interactive understanding of trauma adaptation, emphasizing the need for context-sensitive, theoretically informed approaches in both research and intervention planning.

## 6 Limitations of the study

An important limitation of this study is its cross-sectional design, which restricts strong interpretive claims regarding directionality. Although a serial mediation model was tested, the temporal ordering of psychological resilience, life engagement, coping humor, and post-earthquake trauma cannot be empirically established. Therefore, the findings should be interpreted as reflecting statistically significant associative and indirect relationships that are theoretically consistent with the proposed conceptual framework, rather than as evidence of definitive explanatory processes. Future longitudinal or experimental studies are needed to examine further the temporal sequencing and theoretical processes underlying the relationships among psychological resilience, life engagement, coping humor, and post-earthquake trauma.

The self-report scales employed in this study involve several methodological limitations. First, due to social desirability bias, participants may refrain from disclosing their genuine thoughts, feelings, and behaviors, instead providing responses they perceive as more socially acceptable (Fisher, 1993). This tendency is particularly problematic when addressing sensitive topics, as it can reduce data accuracy (Krumpal, 2013). Moreover, because self-report techniques rely on individuals’ subjective perceptions, cognitive constraints (e.g., recall errors, selective memory, exaggeration), and motivational factors (e.g., the tendency to present oneself in an overly positive or negative manner) can introduce systematic biases (Chan, 2009). In addition, participants’ personality traits, current mood, attentional focus, and the way questions are formulated may all influence response consistency (Paulhus and Vazire, 2007; Podsakoff et al., 2003). Collectively, these factors can restrict the objectivity and internal validity of the findings, thereby limiting the generalizability of the results (Tourangeau and Yan, 2007).

Additionally, the study’s sample was limited to physical education and sport teachers working in disaster-affected areas before and after the earthquake, which may restrict the generalizability of the findings to other occupational groups or geographic regions. Using only a convenience sampling method limited the study to participants accessible to the researchers and constrained the representativeness that could be achieved through random sampling. Conducting data collection via online surveys may have restricted participants’ ability to express their experiences and trauma responses in depth; particularly on emotionally sensitive topics, participants may have been inclined to provide socially desirable responses. Furthermore, the study’s cross-sectional design does not allow for the direct examination of directional or temporal relationships among the variables and captures associations at a single point in time. This limitation may affect the generalizability of the findings.

## 7 Practical recommendations

Training sessions to strengthen psychological resilience, workshops that promote life engagement, and group activities that enhance the functional use of humor can be organized for teachers.

Educational institutions should establish guidance units and psychosocial support centers that facilitate teachers’ integration of these skills into their daily professional lives.

Group work and individual counseling services where teachers can share their experiences should be provided to help reduce post-traumatic stress symptoms.

Enhancing teachers’ psychological resilience is recommended to be integrated into educational policies, as it may indirectly contribute to students’ psychosocial development.

In collaboration with the Ministry of National Education, universities, and local authorities, psychosocial intervention and resilience programs for teachers in disaster-affected regions should be expanded.

### 7.1 Recommendations for future research

Although life engagement functioned as a protective factor in the present model, future studies employing moderation or non-linear analytical approaches may investigate whether different forms or intensities of life engagement produce differential psychological outcomes under prolonged traumatic exposure (Park, 2010).

Future studies could include different occupational groups, students, or family members to enhance the generalizability of the findings.

Instead of cross-sectional designs, longitudinal studies should be conducted to examine how psychological resilience, life engagement, and humor affect trauma over time.

Beyond self-report scales, incorporating observations, in-depth interviews, or biological/psychophysiological measures could provide more objective data.

Conducting similar studies across different cultures may reveal whether the protective role of psychosocial resources is universal or varies according to cultural contexts.

Considering that life engagement may not always serve as a protective factor, future research should further investigate under which conditions it may act as a risk-enhancing factor.

## Data Availability

The raw data supporting the conclusions of this article will be made available by the authors, without undue reservation.
