# Supplementary material for: Resilience after the quake: life engagement and humor as pathways to trauma recovery
Source: Front Psychol. 2026 Feb 26;17:1791041. doi: 10.3389/fpsyg.2026.1791041 (PMC12979233; doi:10.3389/fpsyg.2026.1791041)
Supplement: Supplementary file 1 [file Table_1.pdf]

Appendix A

Exploratory factor analyses of the scales are given below.

Table 1. Factor analysis of the Brief Resilience Scale (BRS)

Item's	The Brief Resilience Scale (BRS)
Item 1	.788
Item 2	.745
Item 3	.890
Item 4	.875
Item 5	.652
Item 6	.878

Table 2. Factor analysis of The Life Engagement Scale (LES)

Item's	The Life Engagement Scale (LES)
Item 1	.799
Item 2	.911
Item 3	.801
Item 4	.685
Item 5	.786
Item 6	.839

Table 3. Factor analysis of The Coping Humor Scale (CHS)

Item's	The Coping Humor Scale (CHS)
Item 1	.945
Item 2	.633
Item 3	.911
Item 4	.744
Item 5	.862
Item 6	.867
Item 7	.844

Table 4. Factor analysis of the Post-Earthquake Trauma Level Determination Scale (PETLDS)

Items	The Post Earthquake Trauma Level Determination Scale (PETLDS)
Item 1	.849
Item 2	.570
Item 3	.698
Item 4	.454
Item 5	.654
Item 6	.535
Item 7	.623
Item 8	.561
Item 9	.806
Item 10	.916
Item 11	.897

Item 12	.938
Item 13	.525
Item 14	.486
Item 15	.437
Item 16	.486
Item 17	.825
Item 18	.796
Item 19	.556
Item 20	.753
